# Delayed anterior cruciate ligament reconstruction and risk of meniscus injury: Exploring the safest delay interval

**DOI:** 10.1002/jeo2.70006

**Published:** 2024-08-27

**Authors:** Ghuna Arioharjo Utoyo, Dliyauddin Fachri, Calvin

**Affiliations:** ^1^ Department of Orthopaedics and Traumatology, Dr. Hasan Sadikin General Hospital Universitas Padjadjaran Bandung Indonesia; ^2^ Faculty of Medicine Universitas Sumatera Utara Medan Indonesia

**Keywords:** clinical outcomes, delayed ACLR, meniscus injury, safest delay interval

## Abstract

**Purpose:**

The duration for which anterior cruciate ligament reconstruction (ACLR) can be delayed without resulting in a risk of subsequent meniscus injury has remained a debatable topic. The main purpose of this study was to determine the safest delay interval for a delayed ACLR.

**Methods:**

This retrospective study included all patients who underwent ACLR between January 2020 and January 2022. The patients were divided into four groups based on the delay interval: <3 months, 3–6 months, 6–12 months and >12 months. Clinical outcomes were assessed using the International Knee Documentation Committee (IKDC) score and Knee Injury and Osteoarthritis Outcomes Score (KOOS) at 1‐year postoperatively.

**Results:**

A total of 95 patients were included in this study. ACLR delay of 3–6 months was not associated with the risk of meniscus injury, while a delay of 6–12 months (odds ratio [OR] = 4.35; 95% confidence interval [CI] = 1.13–16.79; *p* = 0.031) and >12 months (OR = 10.68; 95% CI = 2.55–42.22; *p* = 0.001) was associated with a likelihood of developing meniscus injury. Meniscus injury risk increased by 12% for each month of ACLR delay (OR = 1.12; 95% CI = 1.04–1.22; *p* = 0.003). Regarding clinical outcomes at 1‐year postoperatively, all groups exhibit the same clinical results.

**Conclusion:**

ACLR can be safely delayed up to 6 months after the initial injury. However, a delay for >6 months must be avoided, as it was found to significantly increase the likelihood of developing a meniscus injury.

**Level of Evidence:**

Level III, retrospective comparative study.

**Abbreviations**
ACL: anterior cruciate ligament
ACLR: anterior cruciate ligament reconstruction
IKDC: International Knee Documentation Committee
KOOS: Knee Injury and Osteoarthritis Outcomes Score
RTS: return to sports

## INTRODUCTION

Anterior cruciate ligament reconstruction (ACLR) has been ranked amongst the most commonly performed orthopaedic procedures worldwide. In the United States alone, the rate of ACLR has reached up to approximately 400,000 procedures annually [10, 21]. Similarly, the rate of this procedure in Indonesia is also increasing, with a reported rate of 42% increase in implant usage in 2019 when compared to 2018 [7]. Despite the high volume of ACLR performed globally, longstanding controversy still remains about the optimal timing of intervention. Previously, early intervention (<3 weeks after injury) has been advocated because it was reported that early ACLR resulted in superior postoperative knee function, fewer meniscal procedures and better clinical outcomes when compared to ACLR performed afterwards [3, 5]. However, recent studies found that early ACLR is associated with an increase in the incidence of arthrofibrosis [4]. Agarwal et al. [1] revealed that ACLR delay resulted in a lesser risk of arthrofibrosis requiring surgical intervention. It was found that a delay of at least 6 weeks in patients aged <40 years is associated with a 65% risk reduction, while a delay of 10 weeks in patients aged ≥40 years resulted in a 35% risk reduction. The same findings were reported in the systematic review by Hopper et al. [14]. It was found that early ACLR became one of the factors associated with arthrofibrosis requiring manipulation under anaesthesia or lysis adhesions. Due to those reasons, a delayed ACLR might be an option to prevent such complications. In addition, some evidence has also suggested that both early and delayed ACLR yield the same clinical and stability outcomes [16, 17].

Theoretically, delaying the ACLR may allow an inflammation‐free period, better soft tissue optimization and lower the risk of arthrofibrosis [11]. Unfortunately, the exact duration for how long the ACLR can be delayed still becomes a matter of debate. While some authors reported that the ACLR can be delayed by up to 12 months without posing a significant risk for the development of meniscal damage, others suggest that it should be performed within 3 months to avoid such risk [6, 15, 25]. Therefore, knowing the safest delay interval is crucial, as it helps the surgeon decide the limit of conservative treatment trials that can be done without risking the meniscus for damage. Our main goal in this study is to determine the threshold interval of a delayed ACLR to the occurrence of meniscus injury. We also aim to assess the clinical outcomes of a delayed ACLR at a 1‐year postoperative follow‐up. We hypothesize that (1) a delayed ACLR for >6 months significantly increases the risk of meniscus injury and (2) a delayed ACLR for >6 months resulted in less favourable clinical outcomes compared to ACLR performed ≤6 months.

## MATERIALS AND METHODS

### Study design and patient selection

This is a retrospective study that included all patients who underwent ACLR with a minimal postoperative follow‐up of 1‐year during the period between January 2020 and January 2022. Patients with a previous history of knee trauma prior to current ACL rupture, previous history of meniscal symptoms (pain, clicking, catching, locking), previously diagnosed meniscal injuries or other ligamentous injury, other concomitant knee injuries (fracture, dislocation, other ligaments injury), unknown date of injury and incomplete medical records were excluded from the study. Data included in this study were obtained from electronic medical records.

### Baseline data

Baseline data included in this study consists of age, sex, aetiology of injury (sport‐related, recreational‐related or motor vehicle accidents), injury to surgery time (delay duration), meniscus involvement and type of meniscal procedures (meniscus repair or partial meniscectomy). Patients were categorized according to the delay interval: less than 3 months, 3–6 months, 6–12 months and over 12 months. The delay duration was determined based on the date of initial injury until the date of the ACLR procedure. All ACLR decisions were made by patients without any influence from the surgeon. The surgery was performed by a single sports surgeon, and all of the ACLR procedures were conducted using a hamstring graft. Assessment of meniscus involvement was based on the diagnostic arthroscopy performed during the ACLR.

### Rehabilitation protocol and postoperative follow‐up

Postoperative follow‐up was conducted at first, second, fourth, sixth and 12th months after the initial surgery. At 1 week after the ACLR, all patients were referred to a sports clinic for a rehabilitation protocol. A knee brace was used for 2 months, with a gradual increase of 10° per week for the first 3 weeks, followed by an increase of 15° per week until the brace reached 120°. Knee flexibility exercises were performed during the 2 months postsurgery, followed by muscle strengthening exercises for the next 4 months. The return‐to‐sport rehabilitation protocol was performed at 6 months postoperatively. At a 12‐month postoperative follow‐up, an assessment of residual knee laxity and clinical outcomes was conducted. The knee laxity assessment was performed by the same sports surgeon who had performed the ACLR. Clinical outcomes were assessed using the International Knee Documentation Committee (IKDC) score and Knee Injury and Osteoarthritis Outcomes score (KOOS).

### Statistical analysis

Data analyses were conducted using IBM SPSS Statistics for Windows (Version 26.0; IBM Corporation). Numerical data were presented as mean ± standard deviation (SD). Categorical data were presented as a total number (% percentage). The Kolmogorov–Smirnov test was used to assess the normality of the numerical data. A comparison of characteristics between each group was performed using the Mann–Whitney test for numerical data and the Kruskal–Wallis test for categorical data. Logistic regression was carried out to analyse the factors associated with the risk of meniscus injury. Comparison of knee laxity, IKDC score and KOOS between each group at 1‐year postoperative follow‐up was performed with the Mann–Whitney test. Statistically significant was defined by a *p* value < 0.05.

## RESULTS

**Figure 1:**
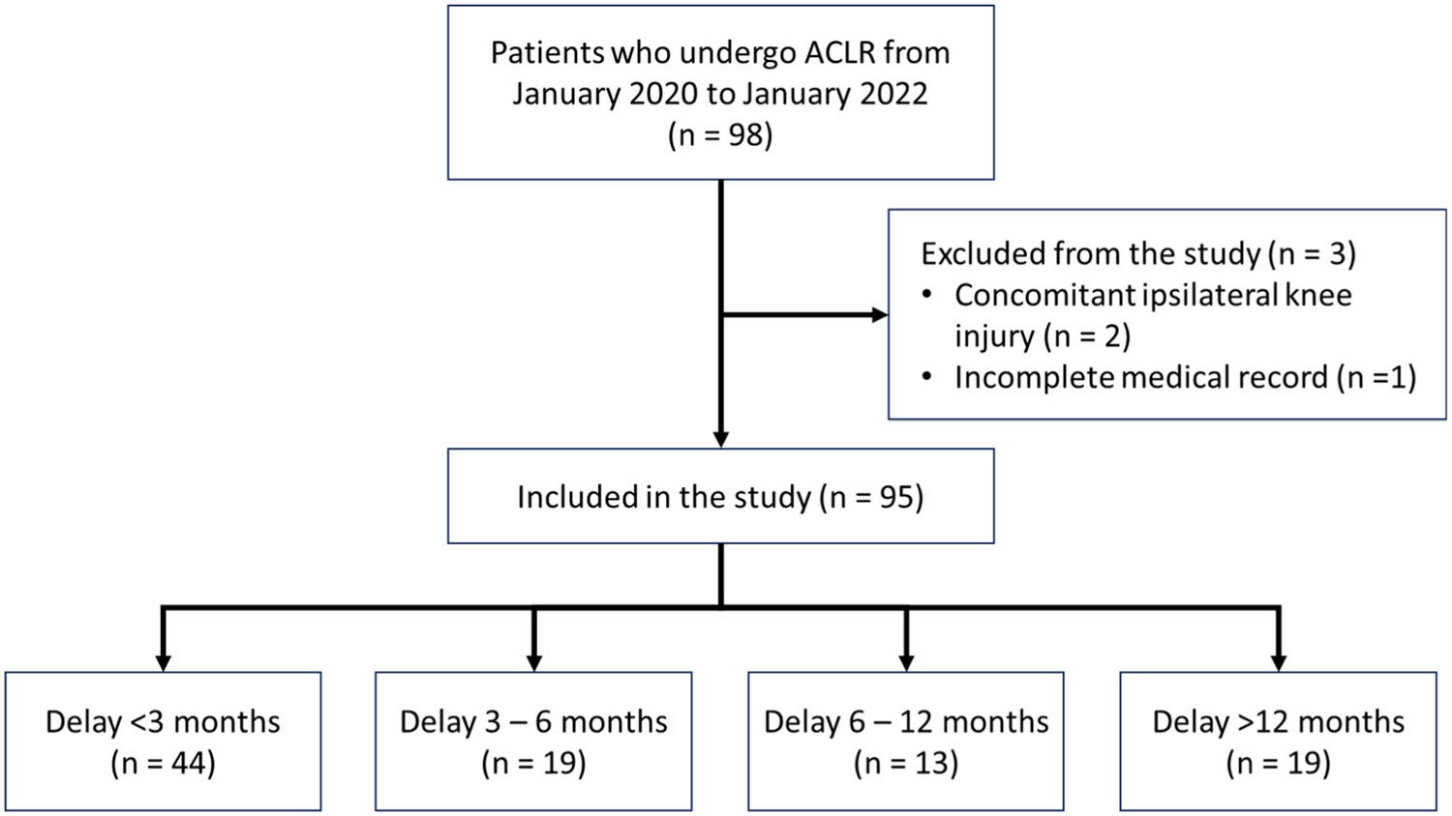
Inclusion and exclusion flowchart of study subjects. ACLR, anterior cruciate ligament reconstruction.

Between January 2020 and January 2022, a total of 98 patients were recorded to undergo ACLR at our institution. However, two patients were excluded due to posterior cruciate ligament involvement and one due to incomplete medical records, leaving only 95 patients included in this study (Figure 1). We categorized the patients into four groups based on the delay interval, consisting of 44 patients (46.32%) in the <3 months group, 19 (20%) in the 3–6 months group, 13 (13.68%) in the 6–12 months group and 19 (20%) in the >12 months group.

Baseline characteristics between each group are summarized in Table 1. Age, gender and aetiology of injury were found to be similar across all groups. The percentage of meniscus involvement was lowest (34.1%) in the group with delay <3 months and the highest (84.2%) in the group with delay >12 months. The difference in the meniscal involvement between each group was found to be statistically significant (*p* = 0.001). Additionally, a significant difference in the rate of meniscal procedures was found between each group (*p* = 0.023). The group with delay <3 months had the highest rate of meniscus repair (20.5%), while the group with delay >12 months had the highest rate of partial meniscectomy (68.4%).

**Table 1:** Baseline characteristics of patients between each group.

Parameter	Delay duration				*p* Value
	<3 months (*n* = 44)	3–6 months (*n* = 19)	6–12 months (*n* = 13)	>12 months (*n* = 19)	
Age	30.36 ± 11.44	28.05 ± 6.22	29.23 ± 7.84	34.58 ± 8.45	n.s.
Gender					n.s.
Male	37 (84.1%)	16 (84.2%)	12 (92.3%)	16 (84.2%)	
Female	7 (15.9%)	3 (15.8%)	1 (7.7%)	3 (15.8%)	
Aetiology					n.s.
Sport‐related	34 (77.3%)	15 (78.9%)	9 (69.2%)	14 (73.7%)	
Recreational‐related	5 (11.4%)	3 (15.8%)	2 (15.4%)	3 (15.8%)	
MVA	5 (11.4%)	1 (5.3%)	2 (15.4%)	2 (10.5%)	
Injury to surgery time (months)	1.11 ± 0.73	4.43 ± 0.75	7.88 ± 1.46	28.56 ± 36.50	<0.001
Meniscus involvement					0.001
Absent	29 (65.9%)	12 (63.2%)	4 (30.8%)	3 (15.8%)	
Present	15 (34.1%)	7 (36.8%)	9 (69.2%)	16 (84.2%)	
Meniscal procedures					0.023
Meniscus repair	9 (20.5%)	2 (10.5%)	1 (7.7%)	3 (15.8%)	
Partial meniscectomy	6 (13.6%)	5 (26.3%)	8 (61.5%)	13 (68.4%)	

Abbreviations: MVA, motor vehicle accident; n.s., not significant.

### Risk of meniscus injury

The factors associated with the risk of meniscus injury are shown in Table 2. A delay of 3–6 months was not found to be associated with a risk of meniscus injury, while a delay of 6–12 months (odds ratio [OR] = 4.35; 95% confidence interval [CI] = 1.13–16.79; *p* = 0.031) and >12 months (OR = 10.68; 95% CI = 2.55–42.22; *p* = 0.001) was found to significantly increase the likelihood of developing meniscus injury. The risk of meniscus injury increased by 12% for each month of ACLR delay (OR = 1.12; 95% CI = 1.04–1.22; *p* = 0.003). In addition, no association was found between age, sex and aetiology to the odds of developing meniscus injury.

**Table 2:** Factors associated with the risk of meniscus injury according to logistic regression analysis.

Variable	OR	95% CI	*p* Value
Delayed ACLR	1.12	1.04–1.22	0.003
Delayed ACLR interval			
<3 months (Reference)	1		
3–6 months	1.20	0.38–3.75	n.s.
6–12 months	4.35	1.13–16.79	0.031
>12 months	10.68	2.55–42.22	0.001
Age	1.01	0.96–1.06	n.s.
Male sex	1.35	0.38–4.81	n.s.
Aetiology			
Sport‐related	1		
Recreational‐related	0.65	0.15–2.87	n.s.
MVA	0.45	0.07–3.14	n.s.

Abbreviations: ACLR, anterior cruciate ligament reconstruction; CI, confidence interval; MVA, motor vehicle accident; n.s., not significant; OR, odds ratio.

### Clinical outcomes at 1‐year postoperative

The comparison of clinical outcomes at 1‐year follow‐up between groups is shown in Table 3. The presence of residual knee laxity was lowest in the group with delay <3 months (11.4%). In addition, both IKDC and KOOS were also found to be highest in the group with delay <3 months. However, the difference between each group was not found to be statistically significant.

**Table 3:** Comparison of clinical outcomes at 1‐year follow‐up between each group.

Outcomes	Delay duration				*p* Value
	<3 months (*n* = 44)	3–6 months (*n* = 19)	6–12 months (*n* = 13)	>12 months (*n* = 19)	
Knee laxity					n.s.
Present	5 (11.4%)	3 (15.8%)	1 (7.7%)	6 (31.6%)	
Absent	39 (88.6%)	16 (84.2%)	12 (92.3%)	12 (68.4%)	
IKDC	84.46 ± 7.87	84.27 ± 3.77	83.82 ± 4.16	80.88 ± 5.76	n.s.
KOOS	83.76 ± 3.61	82.91 ± 3.56	82.18 ± 5.83	81.10 ± 4.51	n.s.
KOOS Sx	87.27 ± 8.10	86.84 ± 5.33	86.15 ± 9.39	84.47 ± 10.66	n.s.
KOOS Pain	84.41 ± 6.03	84.21 ± 6.48	83.33 ± 7.69	82.75 ± 6.39	n.s.
KOOS ADL	91.51 ± 4.40	91.33 ± 7.00	91.40 ± 5.30	90.71 ± 5.21	n.s.
KOOS Sport	76.48 ± 9.56	72.89 ± 12.28	71.15 ± 8.20	71.58 ± 10.42	n.s.
KOOS QoL	79.12 ± 13.74	79.28 ± 14.59	78.85 ± 12.38	75.99 ± 15.35	n.s.

Abbreviations: ADL, activities of daily living; IKDC, International Knee Documentation Committee; KOOS, Knee injury and Osteoarthritis Outcomes Score; n.s., not significant; QoL, quality of life; Sx, symptoms.

## DISCUSSION

The main finding in our study is that an ACLR can be safely delayed for up to 6 months without resulting in the risk of meniscus injury. Meanwhile, each month of ACLR delay was found to increase the risk of developing meniscus injury by 12%. Regarding clinical outcomes at 1 year postoperatively, we found that delayed ACLR of 3–6 months, 6–12 months and >12 months yield the same result as ACLR performed within 3 months.

To date, conflicting evidence still exists regarding the safest delay duration for ACLR. One of the main concerns about delaying an ACLR is the occurrence of meniscus injury. Keyhani et al. [15] have identified that a delay >3 months is already enough to produce the risk of subsequent meniscus injury. On the other hand, Cristiani et al. [6] have reported a much longer threshold of the delay duration. They found that the rate of medial meniscus injury only started to increase for a delay >12 months, while a delay for >6 months only resulted in abnormal knee laxity. Michalitsis et al. [18] revealed that an ACLR delay >12 months did not significantly increase the risk of meniscus injury, which this finding is contrary to both studies above. In addition, it was also reported that the odds of having an isolated medial meniscus tear only increase by 6% each year, while the odds of having a bilateral meniscal tear only increase by 9% each year. In comparison, our study has revealed that there is an increase of 12% for each month of ACLR delay. This can be seen in the increased need for meniscal procedures amongst the 6–12 months and >12 months delay group. Our present study findings indicated that a delay >6 months is associated with the presence of meniscus involvement and the need for meniscal procedures, especially partial meniscectomy.

The timing of ACLR significantly influences the risk of meniscus injury occurrence. The loss of anterior translation resist function caused by deficient ACL leads to an increase in episodes of knee instability, which might further tear the meniscus. A systematic review by Sommerfeldt et al. [22] has established a clear association between recurrent instability episodes and the likelihood of meniscal damage. Therefore, for a patient who wants to delay their surgery, we strongly advise them to limit strenuous knee activity during the delay period. Additionally, the use of a knee brace is advised. Several studies have also supported the use of a brace in an ACL‐deficient knee. A prospective randomized study by Swirtun et al. [23] revealed that a brace reduced an instability episode amongst nonoperated ACL patients. Tomescu et al. [24] also reported that the brace equipped was associated with less meniscal strain. In addition, prereconstruction rehabilitation protocol should also be implemented with a focus on strengthening the hamstring muscles. Adequate hamstring strength serves as a resistance to the anterior translation force and prevents the occurrence of knee instability episodes [8].

Aside from the rate of meniscus injury, the clinical outcomes reported for a delayed ACLR also vary between each study. A study by Forsythe et al. [12] found that ACLR delay >6 months resulted in significantly poorer clinical outcomes when compared to ACLR performed ≤6 months. Upon 1‐year postoperative period, it was revealed that the IKDC score, KOOS pain, KOOS quality of life and KOOS sport were significantly lower in the ACLR delay >6 months group. As in the study from the Swedish National Knee Ligament Registry by Bergerson et al. [3], a delayed group was found to have a significantly lower KOOS at 1‐year postoperative when compared to the early ACLR group (≤1 year) and a conservative group.

Contradictory to the results from previously described studies, our study revealed that even a delay >12 months resulted in similar clinical outcomes as ACLR performed <3 months. Despite the average score was higher in an ACLR < 3 months group, the difference was found to be insignificant. These results are supported by the findings in the KANON trial conducted by Frobell et al. [13]. In their study, early ACLR was not found to result in superior clinical outcomes at a 5‐year postoperative period when compared to optional delayed ACLR. The result from the COMPARE trial [20] also revealed that there was only a slight increase in IKDC score in the early surgical reconstruction group compared to the elective delayed reconstruction group. The differences in IKDC scores between those groups were 5.3 (84.7 vs. 79.4), and clinical relevance of the IKDC score was not found.

We hypothesize that the difference in outcomes compared to the result of previously described studies may be due to differences in the postoperative rehabilitation protocol. Most previous studies do not clearly describe their postoperative rehabilitation programme. At our institution, all patients who undergo ACLR are referred to the sports rehabilitation clinic, where sports medicine physicians and sports physiotherapists directly monitor their progress. Additionally, we always received monthly progress reports on each patient, allowing us to tailor individualized rehabilitation protocols. Papandreou et al. [19] found that three sessions/week and five sessions/week of rehabilitation protocol significantly improve quadriceps strength. As in the study conducted by Villa et al. [27], on‐field rehabilitation for 2–5 days per week was found to lead to a complete functional recovery amongst soccer players. The results from randomized controlled trial study conducted by Elabd et al. [9] also reported that a rehabilitation protocol consisting of five sessions per week significantly improved functional outcomes amongst amateur athletes. Therefore, for patients with low‐ to medium‐knee demand, we advised them to undergo rehabilitation for a minimum of three sessions/week. On the other side, patients with high‐knee demand (athletes) must undergo at least five sessions/week.

The comparable outcomes between each delayed group in our study highlight the importance of postoperative rehabilitation protocol. Return to sports (RTS) must be the main goal for every patient who undergoes a rehabilitation protocol after an ACLR [28]. A systematic review conducted by Glogovac et al. [24] reported that the rate of RTS ranged from 56% to 100%, with the average time ranged from 6.7 to 12 months. Gradual weight‐bearing programmes, pain management, ROM restoration, efforts to decrease the rate of knee effusion and both hamstring and quadriceps muscle strengthening must be done to increase the rate of RTS [2]. Della et al. [26] have found that high compliance with rehabilitation protocol significantly influenced the RTS rate amongst patients who underwent ACLR revision. Those findings imply that a surgeon must be able to ensure the proper rehabilitation programme for each patient. The success of the rehabilitation programme requires collaboration between the sports surgeon, sports medicine physician and sports physiotherapist.

The main strength of this study is that all patients were managed only by a single sports surgeon, which can minimize the risk of bias. In addition, follow‐ups were also performed by the same sports surgeon. However, this study has several limitations that need to be noted. First, due to the retrospective nature of this study, selection biases may be present. Second, as it is a single‐centre study, the number of subjects included in this study is still limited. Third, we could not provide the information and analyse the risk of cartilage involvement, the specific meniscus location and the severity of meniscus involvement, as it is not a routine practice to include that information in the electronic medical records. Lastly, the knee laxity assessment was only determined clinically through the Lachman test, which results can be prone to bias. Although the findings in the study indicated that a delayed ACLR offers the same clinical outcomes as an ACLR performed within 3 months, a longer follow‐up is still needed to determine the long‐term outcomes of delayed ACLR and the association of a delayed ACLR with the risk of cartilage damage and early knee osteoarthritis.

## CONCLUSION

The safest threshold for a delayed ACLR is up to 6 months after the initial injury. Performing the ACLR afterwards will significantly increase the likelihood of developing subsequent meniscus injury. In terms of outcomes, a delayed ACLR > 12 months results in a similar clinical outcome at the 1‐year period as ACLR performed within 3 months.

## AUTHOR CONTRIBUTIONS

All authors have made substantial contributions to the conception and design of the study, acquisition of data, analysis and interpretation of data, drafting the article or revising it critically for important intellectual content and final approval of the version to be submitted. Each of the authors has read and concurs with the content in the final manuscript.

## CONFLICT OF INTEREST STATEMENT

The authors declare no conflict of interest.

## ETHICS STATEMENT

The research was approved by the Ethics Committee of Dr. Hasan Sadikin General Hospital Bandung (ID number of study approval: DP.04.03/D.XIV.6.5/160/2024). Informed consent was obtained from all patients included in this study.

## Data Availability

The data that support the findings of this study are available from the corresponding author upon request.
